# Coronary heart disease and stroke disease burden attributable to fruit and vegetable intake in Japan: projected DALYS to 2060

**DOI:** 10.1186/s12889-019-7047-z

**Published:** 2019-06-07

**Authors:** Xiuting Mo, Ruoyan Tobe Gai, Kimi Sawada, Yoshimutsu Takahashi, Sharon E. Cox, Takeo Nakayama, Rintaro Mori

**Affiliations:** 1 Department of Health Policy, National Center for Child Health and Development, Okura 2-10-1, Setagaya-ku, Tokyo, 157-8535 Japan; 2 Department of Health Informatics, Kyoto University School of Public Health, Yoshidakonoe cho, Sakyo Ward, Kyoto, Kyoto Prefecture 606-8501 Japan; 3 Department of Empirical social Security Research, National Institute of Social Security and Population Research, Uchisaiwaicho 2-2-3, Chiyoda-ku, Tokyo, 1000011 Japan; 4 Faculty of Human Life and Environmental Sciences, Nagoya Women’s University, 3 Chome-40 Shiojicho, Mizuho Ward, Nagoya, Aichi Prefecture 467-0003 Japan; 5 School of Tropical Medicine & Global Health, Nagasaki University, 1-14 Bunkyomachi, Nagasaki, 852-8521 Japan; 6 Faculty of Epidemiology & Population Health, London School of Hygiene & Tropical Medicine, London, UK

**Keywords:** Fruit and vegetable, Nutrition, Cardiovascular disease, DALY preventable fraction, Prediction

## Abstract

**Background:**

Fruit and vegetable consumption was considered a protective effect against cardiovascular and cerebrovascular diseases (CVDs). This study aimed to project the reduction in the CVD burden under different scenarios of increased fruit and vegetable intake in Japan by 2060.

**Methods:**

Population attributable fractions (PAF) were calculated by gender and age in 2015. The projection considered five scenarios for 2015, 2030, 2045, and 2060: 1) a baseline of no changes in intake; 2) a moderate increase in fruit intake (extra 50 g/day or 1/2 serving); 3) an high increase in fruit intake (extra 100 g/day or 1 serving); 4) a moderate increase in vegetable intake (extra 70 g/day or 1 serving); and 5) an high increase in vegetable intake (extra 140 g/day or 2 servings). Potentially preventable disability-adjusted life years (DALYs) for CVDs were estimated for each scenario. Monte Carlo simulations were performed to calculate the 95% confidence intervals of the estimates.

**Results:**

Across all age groups, men had a higher daily vegetable intake than women (292.7 g/d > 279.3 g/d) but a lower daily fruit intake (99.3 g/d < 121.0 g/d). Comparing with recommended intake level (350 g/d of vegetable and 200 g/d of fruit), the total CVD burden was estimated to be 302,055 DALYs attributable to inadequate fruit consumption in 2015, which accounted for 12.6% of the total CVD burden (vegetable: 202,651 DALYs; 8.5%). In 2060, the percentage of the CVD burden due to insufficient intake of fruit is estimated to decrease to 7.9% under the moderate increase scenario and to decrease to 4.5% under the high increase scenario (vegetable: 5.4%; 2.4%).

**Conclusions:**

The study suggested that a relevantly large percentage of the CVD burden can be alleviated by promoting even modest increases in fruit and vegetable consumption in Japan.

**Electronic supplementary material:**

The online version of this article (10.1186/s12889-019-7047-z) contains supplementary material, which is available to authorized users.

## Background

The association between fruit and vegetable intake and the risk of chronic diseases including cardiovascular and cerebrovascular diseases (CVD) has been confirmed by previous epidemiological studies [1–3]. In Japan, CVD are among the top contributors to the disease burden [4], accounting for over 20% of the total medical expenditure [5]. Over the past 50 years, mortalities due to CVD in the aging population have declined, especially for those 80 years old or older. The mortality rate due to cardiovascular diseases among individuals in their 80’s decreased from 2.24% in 1965 to 0.91% in 2017 while the mortality rate among those in their 90’s decreased from 4.79% in 1965 to 2.96% in 2017 [6, 7]. However, the CVD burden is expected to increase among the rapidly aging population of Japan [8].

The consumption of vegetables and fruits is lower among adults in Japan than in other countries [9]. In Japan, a minimum of 350 g/d of vegetable (5 servings at 70 g per serving, not including potato or legumes) and 200 g/d of fruit (2 servings at 100 g per serving) per day are recommended [9]. The UK’s recommendation is 5 portions/d (400 g/d) of fruits and vegetables (not including potato or legumes) [10]; the US recommends 2~3 cups/d of vegetables (168 g/d ~ 252 g/d, depending on age and sex) and 1~2 cups/d of fruit (depending on age, sex, and level of physical activity) for individuals who get less than 30 min per day of moderate physical activity [11]. Recent results of the Japanese National Health and Nutrition Survey 2016 indicate that in adults over 20 years old, the daily consumption remains as low as 269.4 g for vegetables and 98.9 g for fruits (adjusted by age) [12] and that there has been a significant decline from 2006 (vegetables: 300.5 g/d; fruits: 107.5 g/d) [13].

The aim of this study was to estimate and project the CVD burden at different scenarios of vegetable and fruit intake and to simulate the potential impact on CVD burden in Japan by improving vegetable and fruit consumption in a long term.

## Methods

### Data sources

We used data from the 2015 Japanese National Health and Nutrition Survey (NHNS), the 2017 Japanese Patients survey, and 2017 Japanese National Institute of Population and Social Security Research.

The NHNS is a random, stratified sampling survey conducted annually by the Japanese Ministry of Health, Labour and Welfare in order to understand the status of people’s health, nutritional intake, and lifestyle habits and to obtain basic data necessary to promote public health comprehensively. The mean value for daily fruit and vegetable consumption by sex and age group derive from this study [13].

The Japanese Patients Survey is a triennial, national, random, stratified sampling survey designed to collect patient data from hospitals and clinics (hereafter referred to as “medical institutions”) including final diagnoses using ICD10 codes and patient outcomes for health policy formulation. From this survey we obtained the number of patients and mortalities due to coronary heart disease (CHD, I20-I25) and stroke (I60-I69) in 2014 [8] and calculated the rates.

The population projections for Japan were based on data from the Japanese National Institute of Population and Social Security Research, which projected the size and structure of the population based on assumptions about future mortality and international migration levels to 2065 [14]. We used the population projections to calculate disease prevalence and mortality according to the following formula: prevalence/mortality rate by age group and sex × population projection.

### Diseases estimated

We used coronary heart disease (CHD, I20-I25) and stroke (I60-I69) as two examples of CVD. In Japan in 2014, there were 750,000 estimated cases of CHD accounting for 38.7% of heart disease cases and 1,668,000 estimated cases of CVD accounting for 65.8% of cerebrovascular disease cases (2,534,000) [8]. The number of patients with a CHD was calculated as the sum of patients with angina pectoris and patients with acute myocardial infarction to calculate the Disability Adjusted Life Years (DALY). The number of CHD cases in 2014 was 1,659,000 or slightly lower than the real burden of 1,668,000.

### Fruit/vegetable consumption

Data on fruit and vegetable consumption, drawn from the NHNS 2015, [12] were expressed as ordinal categorical variables by gender (male or female) and age (20–29, 30–39, 40–49, 50–59, 60–69, 70-). Fruit intake (gram/d) was expressed as 0, 1–49, 50–99, 100–149, 150–199, 200–249, 250–299, 300–349, 350–399, and 400-. The percentage of individuals with fruit intake exceeding 200 g was low, particularly in groups younger than 60 years old or less than 10% of the total. Thus, we combined those with an intake greater than 200 g into one group. Vegetable intake (gram/d) was measured as 0–69, 70–139, 140–209, 210–279, 280–349, and above 350.

### Risk ratios (of insufficient fruit/vegetable intake)

We conducted a systematic search of PubMed using “((“fruit”[MeSH Terms] OR “fruit”[All Fields]) AND intake[All Fields]) OR ((“vegetables”[MeSH Terms] OR “vegetables”[All Fields] OR “vegetable”[All Fields]) AND intake[All Fields]) AND ((“cardiovascular system”[MeSH Terms] OR (“cardiovascular”[All Fields] AND “system”[All Fields]) OR “cardiovascular system”[All Fields] OR “cardiovascular”[All Fields]) OR (“stroke”[MeSH Terms] OR “stroke”[All Fields]) OR (“coronary disease”[MeSH Terms] OR (“coronary”[All Fields] AND “disease”[All Fields]) OR “coronary disease”[All Fields] OR (“coronary”[All Fields] AND “heart”[All Fields] AND “disease”[All Fields]) OR “coronary heart disease”[All Fields])) AND (“japan”[MeSH Terms] OR “japan”[All Fields])” on January 19, 2018. We retrieved 97 papers. After abstract screening, 15 studies which were deemed to be related to fruit and vegetable intake & CVDs risk in Japan remained [15–29]. After a full reading of the papers, only nine reporting the risk ratio/hazard risk for “fruit and vegetable intakes & CVDs risk in Japanese populations” (Additional file 1: Table S15) were found [21–29]. Data extraction was conducted by Mo and was checked for accuracy by Gai. However, the measurement of fruit and vegetables intake was not standardized among the studies. Seven used intake quantiles, but units of measurement differed including: gram/1000 kcal [24, 25], gram/day [23, 27, 28], servings/week [22], and servings/day [23]. The remaining two studies used intake frequency [21, 29]. We decided it was not possible to calculate dose-response relative risk based on these Japanese studies; therefore, we conducted a second systematic search of the literature for existing meta-analyses of CVD risk and fruit and vegetable intake not limited to the Japanese population. We searched PubMed and Embase using the keywords, “systematic[sb] AND ((((fruit intake) OR vegetable intake) AND (cardiovascular OR stroke OR (coronary heart disease)))) AND meta” for publications between 2017/01/01 to 2018/01/19. As a result, we identified 15 publications, which on further review was limited to two relevant meta-analyses [1, 2]. Both studies used PubMed and Embase. One study included 142 publications up to July 19, 2016 (54 on CHDs, 43 on strokes, 48 on CDVs, and 39 on cancer) based on 95 cohort studies [2] while the other study was less recent (June 2014) and included 38 studies based on 47 cohorts [1]. The RR calculated in the former study separated CVD into CHD and stroke; a dose-response analysis also listed more groups based on a 50 g/d difference. Therefore, we used the risk ratios calculated in the former meta-analysis, which included a greater number of reports as well as the latest research.

### Calculation of population attributable fraction (PAF)

The prevalence of CVDs and CVD-caused mortality (CHD and stroke combined) attributable to low fruit and vegetable intake by sex and age were estimated based on the population attributable fraction (PAF) according to the definition of the World Health Organization (WHO): PAF = $\mathrm{PAF}=\frac{\sum_{i=1}^n{P}_i{RR}_i-{\sum}_{i=1}^n{P}_i^{\hbox{'}}{RR}_i}{\sum_{i=1}^n{P}_i{RR}_i}$ (P_i_: proportion of the population at exposure level i, the current exposure; P^’^_i_: proportion of population at exposure level i, the counterfactual or ideal level of exposure; RR: the relative risk at exposure level i; n: the number of exposure levels) [30]. P_i_ or P^’^_i_ was calculated from the insufficient intake of fruit and vegetable. The recommend amounts of 200 g/d of fruit intake and 350 g/d of vegetable intake were considered to be sufficient (unexposed level).

### Disease burden

The disease burden in 2015, 2030, 2045, and 2060 was reported in Disability Adjusted Life Years (DALYs) using the formula below: DALY(c, s, a, t) = YLL(c, s, a, t) + YLD (c, s, a, t) for a given cause c, age a, sex s, and year t [31]. YLLs (years of life lost) = N × L, where N is the number of deaths and L is the standard life expectancy at the age of death in years. YLDs (years lost due to disability) = P × DW where *P* is the number of prevalent cases and DW is disability weight. The mortality and prevalence data were drawn from the Patient Survey (2015) [8]; population life expectancy and projection data were drawn from the Japanese National Institute of Population and Social Security Research [14]; DW data were drawn from Japanese studies and the WHO Global burden disease report [32–34]. For details, please refer to Additional file 1: Tables S13-S14.

### Simulation of the long-term impacts

In the projection, we considered five scenarios, including: 1) the base scenario of no change in consumption, with 18.3 and 30.3% consuming more than the recommended amount of fruit and vegetable, respectively; 2) moderate increase in fruit intake defined as an increase in the consumption of fruit by 50 g/day (1/4 of the recommended amount), with 27.4% of the population consuming more than the recommend daily allowance; 3) an high fruit intake scenario defined as an increase in the consumption of fruit by 100 g/day (2/4 of the recommended daily allowance), with 39.0% of the population consuming more than the recommended daily allowance of fruits; 4) a moderate increase in vegetable intake defined as an increase in the consumption of vegetable by 70 g/day (1/5 of the recommended daily allowance), with 44.3% of the population consuming more than the recommended amount; and 5) an high vegetable intake scenario defined as an increase in the consumption of vegetables by 140 g/day (2/5 of the recommended daily allowance), with 62.1% of the population consuming more than the recommend amount of vegetable. Monte Carlo simulation was performed (1000 iterations) using EXCEL to calculate the 95% confidence intervals (95% CIs) for the estimated DALY.

## Results

### Current fruit/vegetable consumption (the base scenario)

**Fig. 1:**
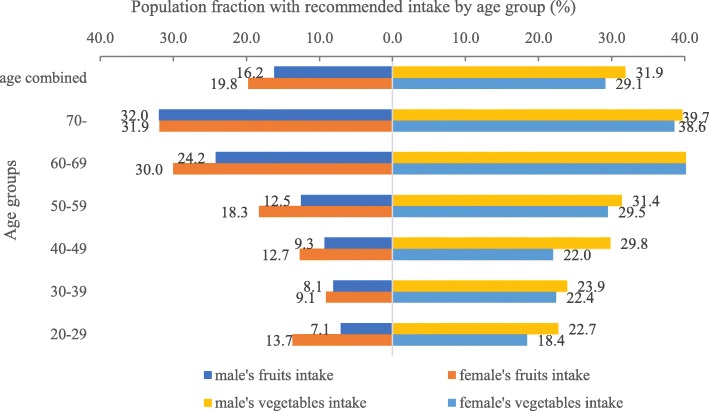
Population fraction with recommend amount of current fruit or vegetable intake by age and gender in 2015 (%)

**Fig. 2:**
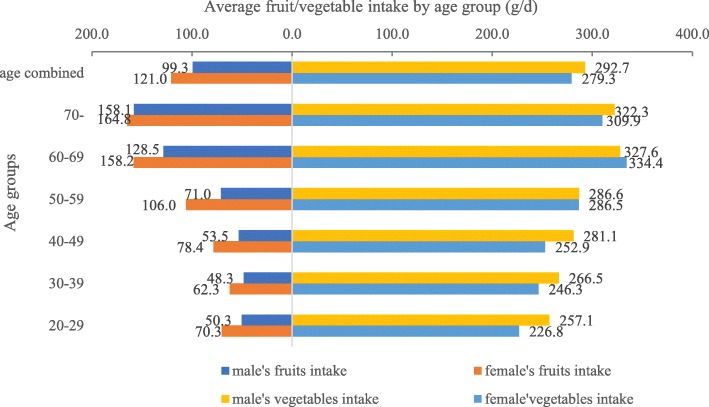
Average fruit/vegetable intake by age and gender in 2015 (g/d)

Both fruit and vegetable intake increased with age, with 10.3, 8.6, 11.0, 15.4, 27.2, and 32.0% in different age groups (20–29 years, 30–39 years, 40–49 years, 50–59, years, 60–69 years, and 70+ years, respectively) consuming more than the daily recommended amount of vegetable, and 20.1, 23.2, 25.9, 30.4, 40.4, and 39.1% of the respective age groups consuming more than the recommended amount of fruit (Fig. 1). Across all age groups, men had a higher daily vegetable intake (292.7 g/d > 279.3 g/d) but lower daily fruit intake (99.3 g/d < 121.0 g/d), with the greatest difference appearing in the youngest age group. For all age groups combined, 31.9% of men consumed more than the daily recommended intake of vegetable while 16.2% consumed more than the daily recommended amount of fruit compared to 29.1 and 20.0% for vegetable and for fruit, respectively, in women. The details of the average fruit and vegetable intake by age and gender are shown in Fig. 2.

### Population attributable fraction (PAF) for current fruit and vegetable intake under different scenarios

Table 1 shows the age population weighted average PAF for mortality and prevalence of CVD given the current intake of fruit and vegetable by sex in the different scenarios. The PAF for mortality and prevalence of CVD showed the same trend as intake (see Table 1**)** while the smaller PAF correlated with a lower CVD burden due to fruit and vegetable intake. The PAF for mortality and prevalence were higher in males than in females, but the opposite was true for vegetable intake.

**Table 1:** PAFs (age population weighted average) for mortality and prevalence of CVDs for current intake of fruit and vegetable by gender in 2015

PAFs	Fruits intake				Vegetables intake			
	male	95%CI	female	95%CI	male	95%CI	female	95%CI
Base								
Mortality	18.7	18.5–19.0%	15.8%	15.5–16.0%	10.1%	9.9–10.3%	10.5%	10.3–10.7%
Prevalence	12.0%	11.9–12.0%	10.1%	10.1–10.2%	4.2%	3.9–4.5%	4.3%	4.0%-4.6
Medium								
Mortality	13.4%	13.1–13.7%	10.9%	10.6–11.1%	6.4%	6.2–6.6%	6.7%	6.5–6.9%
Prevalence	9.7%	9.6–9.8%	7.8%	7.7–7.8%	2.8%	2.4–3.1%	2.8%	2.4–3.1%
High								
Mortality	8.6%	8.3–8.9%	6.7%	6.4–7.0%	3.5%	3.3–3.8%	3.7%	3.4–3.9%
Prevalence	5.9%	5.8–6.0%	4.5%	4.4–4.6%	1.4%	1.0–1.7%	1.6%	1.2–1.9%

### Projections of CVDs DALYs under different scenarios until 2060

**Fig. 3:**
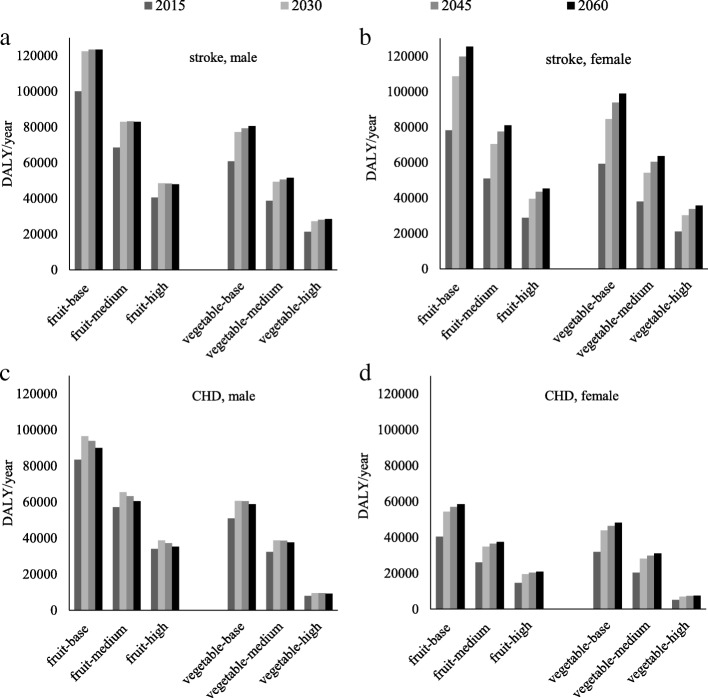
**a** Stroke, male **b** stroke, female **c** CHD, male **d** CHD, female. Stroke, CHD disease burden (DALY) and predictions for insufficient vegetable and fruit intake intake (fruit-base: the base case of no change in consumption; fruit-medium: the consumption of fruit increased by 50 g/day; fruit-high: the consumption of fruit increased by 100 g/day; vegetable-medium: the consumption of vegetable increased by 70 g/day; vegetable-high: the consumption of vegetable increased by 140 g/day)

The overall number of patients with a CVD in 2014 was 1,659,000, comprising 616,000 CHD and 1,043,000 stroke patients. The number of CVD patients was estimated to increase until 2045 (2030: 2,075,710 cases; 2045: 2,211,402 cases), then stabilize in 2060 (2,223,195 cases). The same trend in the projection of DALY caused by stroke and CHD was observed. Details of by gender and sub-diseases are shown in Fig. 3**.** In general, the DALY of males were higher than that of females. The proportions of the CVD disease burden attributable to varying levels of fruit and vegetable intake are shown in Table 2. It was clear that the proportion decreased when intake increased. Approximately 302,055 (12.6%) and 202,651 (8.5%) of DALY from CVD in 2015 were associated with fruit and vegetable intake below the recommended amount. Under the scenario of a moderate increase in fruit and vegetable consumption, the numbers decreased to 8.5 and 5.4%, respectively. Under the scenario of a high increase in fruit and vegetable consumption, the PAF decreased to 4.9 and 2.3%, respectively.

**Table 2:** DALY from CVD and percentages due to lower-than-recommend amount fruit and vegetable intake in 2015, 2030, and 2060

Times New Roman	2015	2030	2060
Original DALYs from CVDs	2,388,489	3,126,036	3,310,571
DALY% associated with vegetable intake			
Base case scenario	202,651 (8.5%)	265,950 (8.5%)	280,060 (8.5%)
Medium increase scenario	129,371 (5.4%)	170,245 (5.4%)	179,417 (5.4%)
High increase scenario	55,425 (2.3%)	73,941 (2.4%)	78,501 (2.4%)
DALY% associated with fruit intake			
Base case scenario	302,055 (12.6%)	381,672 (12.2%)	394,010 (11.9%)
Medium increase scenario	202,536 (8.5%)	253,671 (8.1%)	260,433 (7.9%)
High increase scenario	117,789 (4.9%)	146,212 (4.7%)	149,205 (4.5%)

## Discussion

Based on convincing evidence of an inverse association between fruit and vegetable intake and the CVD rate [17, 22, 27], our results demonstrated a probable reduction in the CVD burden if even a modest improvement in fruit and vegetable intake could be achieved, such as an additional 1/2 serving of fruit or additional 1 serving of vegetable (the moderate increase scenario). The proportion of the CVD disease burden attributable to fruit intake was projected to decrease from 12.6% (the base scenario) to 8.5% (the moderate increase scenario) and to 4.9% (the high increase scenario), and that to vegetable intake from 8.5 to 5.4% and to 2.3%.

Our results are in accordance with previous analyses showing a potentially great impact in reducing cardiovascular disease burden by increasing fruit and vegetable intake [35–37]. While detailed disease burden attributed to inadequate fruit and vegetable intake was divergent due to 1) other studies considered the total effects of fruit and vegetable intake rather than separating their impacts [35–37]. We consider it inappropriate to directly sum up the disease burden, as the risk ratios used in this study were separated and possible overlapping impacts due to double counting if sum up directly; 2) different average daily consumption in heterogenous populations: for example even lower fruit and vegetable intake level in South African population (235 g/d for males and 226 g/d for females) compared to that in Japan (adjusted by age: 372 g/d for males and 400.3 g/d for females) [12, 13], which can partly explain the higher PAF in a simulation study with a South African population (ischemic heart disease PAF: 34.6%; ischemic stroke PAF: 22.2%) [37]; and 3) different theoretical minimum risk of ‘adequate’ intake. For example, some studies chose an intake of 600 g/d in adults as the optimal consumption [35–37] while we used 350 g/d of vegetable and 200 g/d of fruit as recommended by the Japanese government [9].

In addition, daily consumption of both vegetable and fruit remains relevantly low in Japan [25], and the consumption pattern diversifies by age and gender. The national average vegetable and fruit intake in Japan ranked 77th and 133th among 172 countries in 2013, respectively, and was equivalent to about 50% of that in other high income countries [38]. Similar to the tendency in other developed countries, younger people tend to be less likely to consume fruit compared to the older people [35]. On the other hand, different to other researches [35–37], average consumption in female is larger than that in male, especially that of fruit. Such a tendency may be explained by the selective preference to foods: females were more likely to be concerned about freshness and food safety while males were more likely to select foods based on their taste preferences [39].

The projected number of patients with a CVD as well as the DALY due to low fruit and vegetable intake were estimated to increase in future decades (Fig. 3); especially, there is a steady growth shown in the disease burden of female stroke (Fig. 3b). The potential reasons might be a steadily projected increase of female stroke cases from 526,000 in 2014 to 874,797 in 2060; and the majority of the CVD disease burden (> 90%) in Japan is within the growing older population (> 60 years). [12] According to the Patients Survey in 2015: about 94.9 and 86.8% mortality cases happened in female and male group aged above 65 years, respectively. In addition, population projection in Japan showed that the age group above 65 years has a soaring increase from 2015 to 2020, 2030 and 2060 (female: 29.7, 32.1, 34.9, 43.5%; male: 23.8, 25.9, 28.0, 36.1%, respectively) [14].

As a forefront super-aged society, CVD burden is expected to continuously increase in the upcoming decades. The Japanese government initiated the Healthy Japan 21 Project in 2002 (2nd phase from 2013), recommending increased consumption of vegetables and fruit to prevent lifestyle-related diseases [40]. However, vegetable consumption has actually decreased in the last decade from 300.5 g/d in 2006 to 269.4 g/d in 2016 [12] [13]. ,possibly due to a combination of factors: an increasing preference for a western style of diet, characterised by energy dense, processed foods; increased cost, particularly impacting the younger generation with lower purchasing power [41]; and changes in lifestyle resulting in an increased desire/need for foods requiring less preparation time and having a longer shelf-life (from suppliers and individuals), and reduced supply [42]. Although overall fruit consumption has remained stable from 107.5 g/d/person in 2006 to 98.9 g/d/person in 2016 [43] the proportion of fresh fruit consumed has decreased possibly due to increasing prices, ready availability of processed foods or reduced supply and/or accessibility of fresh produce [43]. The number of green grocers fell by half between 2003 and 2014 in tandem with a decrease in areas under cultivation and the yield of fruit trees (statistics from 1975 to 2016) [43]. Decreased fresh fruit and vegetable production in Japan is associated with the aging population and increased urbanization, with the number of farming households decreasing from 510,000 in 2005 to 370,000 in 2015 [41] [43]. ,Global climate change is also likely to be impacting fruit and vegetable production in Japan. Increased CO_2_ concentrations may have a predominantly positive effect on yield but negative effects on nutritional quality while the increased incidence of severe weather events often cause catastrophic damage to crops [44].

The “Dietary Guidelines for the Japanese” was first announced in 2000. In 2005, an upside-down pyramid, the so-called, “Japanese Food Guide Spinning Top” along with the Basic ‘Shokuiku’ (dietary education’) Act was published to promote nutritional education at the community level [45]. This program aimed to teach the Japanese what a well-balanced diet per day should contain: 5–7 servings of grain (rice, bread, noodles, and pasta), 5–6 servings of vegetables, 3-5servings of fish or meat (meat, fish, egg, and soy-beans), 2 servings of dairy (milk and milk products), and 2 servings of fruit [46]. The lack of knowledge among the general population of how the recommended daily servings translate to actual dietary intake was evidenced in one survey of 300,000 adults in 2012, in which over a half of the respondents stated that they did not know the daily recommended intake in grams or how the daily recommended intakes translated into servings [41, 47]. Promoting adequate fruit and vegetable consumption is a significant public health challenge.

On the other hand, although abundant evidence mainly from observational studies showed fruit and vegetable consumption has a protective effect again CVD [1–3], few intervention trials were long enough to examine the effects of increased fruit and vegetable consumption on CVDs without confounders driven from other dietary patterns and lifestyle modifications [48, 49]. Factors contributing to CVDs are complicated, not limited to vegetable and fruit consumption.

### Study limitations

Despite using the best available data resources to calculate the risk ratios associated with fruit and vegetable intake based on a recently published meta-analysis, some limitations remain.

**First**, the risk ratio of fruit/vegetable intake to the risk of CVD was not specific to the Japanese population and may therefore not accurately reflect the different genetics, dietary habits (e.g. more fish, less meat), and physical exercise levels of the Japanese population. **Second**, the RR used in this study did not take each subtype of fruit and vegetable into account. Obviously, different combinations may lead to different effects. Various studies have reported on fruit and vegetable subtypes and the risk of CVDs [2], and not every subtype showed an inverse association with CVD risk. For example, an high intake of items like apples/pears, citrus fruits, fruit juice, green leafy vegetables, and pickled vegetables might decrease the total risk of stroke [2]; however, an high intake of apples/pears, citrus fruits, fruit juice, green leafy vegetables, tomatoes, beta-carotene rich items, and vitamin C rich items had an inverse association with the risk of coronary heart disease [2]. Items such as berries, citrus fruit juices, dried fruits, grapes, canned fruits, strawberries, broccoli, etc. did not show this association with CVD [2]. **Third**, the average energy adjusted fruit/vegetable intake (format of g/1000Kcal) would be optimal but was not used due to lack of suitable data and for the sake of simplicity. Further, data of dietary exposure is from dietary questionnaire assessments [13], reflecting limitation of dietary ascertainment [50].

**In addition,** a greater intake of fruit and vegetables does not necessarily result in more benefits. Some fruits containing high levels of carbohydrates and sugars can increase the blood glucose level, posing a danger to patients with type-2 diabetes [51]. An high intake of pickled vegetables containing large concentrations of N-nitroso compounds might double the risk of esophageal cancer [52, 53]. In this study, the data source on fruit intake included figures on fresh fruit and juice but did not include fruit preserves. Vegetables were categorized as yellow-green vegetables, other vegetables, vegetable juice, and Japanese pickles [54].

The CHD, stroke prevalence, mortality rate, and the life table of the general population used as important estimation indices in this study showed a lower disease burden than the findings of the World Health Organization – Global Burden of Disease Project (WHO-GBDP). The reason might be that, in this study, the disability weight of CHD (ischemic heart disease) only considered acute myocardial infarctions and angina pectoris based on available data; the “standard expected years of life lost” for calculating YLL in this paper used population projections for Japan based on the Japanese National Institute of Population and Social Security Research for age 0 to 105 years [24]. The “standard expected years of life lost” in the WHO-GBDP estimates were age ranging from neonatal to 85+ at 5-year intervals, leading to an higher life expectancy and disease burden (due to greater longevity) for those aged over 85 years. When dealing with the disease data from the official website, we omitted patients with an unknown age to avoid underestimation; fortunately, however, these patients contributed few data points (1/782 in CHD and 1/1046 in stroke).

## Conclusions

This study assessed the long-term CVD burden if increased vegetable and fruit consumption could be achieved and thus aimed to assess the potential impact of nutrition-promoting interventions on public health. Such estimates can be applied in the evaluation of outcomes in public health and nutrition interventions worldwide.

## Additional file

Additional file 1: **Table S1**-**12**: Prevalence population attributable fraction by sex and age. **Table S13**-**14**: Disability weight used in the study. **Table S15**: General information fo selected studies used in the study.(DOCX 252 kb)

## Data Availability

All the original data is publicly accessible from Japanese official website database as reference. Appendix calculation materials are available in https://pan.baidu.com/s/1XY2x-uwywPmvmb2InvKALQ.

## Abbreviations

CHD: Coronary heart disease
CVDs: Cardiovascular and cerebrovascular diseases
DALYs: Disability adjusted life years
NHNS: Japanese National Health and Nutrition Survey
PAF: Population attributable fraction
YLDs: Years lost due to disability
YLLs: Years of life lost
