# Supplementary material for: Coronary heart disease and stroke disease burden attributable to fruit and vegetable intake in Japan: projected DALYS to 2060
Source: BMC Public Health. 2019 Jun 7;19:707. doi: 10.1186/s12889-019-7047-z (PMC6555950; doi:10.1186/s12889-019-7047-z)
Supplement: Supplementary file 1 — Table S1-12: Prevalence population attributable fraction by sex and age. Table S13-14: Disability weight used in the study. Table S15: General information fo selected studies used in the study.(DOCX 252 kb) [file 12889_2019_7047_MOESM1_ESM.docx]

Table 1.1 Prevalence population attributable fraction due to insufficient fruit intake by sex and age group

| age | fruit | | fruit(+50g) | | | | fruit(+100g) | | | |
| --- | --- | --- | --- | --- | --- | --- | --- | --- | --- | --- |
|  | male | female | male | % | female | % | male | % | female | % |
| 20-29 | 0.149 | 0.133 | 0.126 | 0.152 | 0.110 | 0.175 | 0.080 | 0.463 | 0.068 | 0.488 |
| 30-39 | 0.145 | 0.132 | 0.121 | 0.161 | 0.105 | 0.199 | 0.076 | 0.473 | 0.063 | 0.521 |
| 40-49 | 0.145 | 0.128 | 0.122 | 0.159 | 0.102 | 0.200 | 0.077 | 0.470 | 0.062 | 0.512 |
| 50-59 | 0.131 | 0.106 | 0.107 | 0.184 | 0.081 | 0.242 | 0.066 | 0.500 | 0.046 | 0.563 |
| 60-69 | 0.100 | 0.077 | 0.076 | 0.233 | 0.053 | 0.315 | 0.045 | 0.553 | 0.029 | 0.631 |
| 70- | 0.081 | 0.078 | 0.059 | 0.273 | 0.055 | 0.296 | 0.033 | 0.594 | 0.030 | 0.608 |

Table 1. 2 Prevalence population attributable fraction due to insufficient fruit intake by sex and age group, 95% CI

| fruit(+50g)-PPAF | | | | fruit(+100g)-PPAF | | | |
| --- | --- | --- | --- | --- | --- | --- | --- |
| male | | female | | male | | female | |
| lower | upper | lower | upper | lower | upper | lower | upper |
| 0.146 | 0.159 | 0.167 | 0.182 | 0.393 | 0.533 | 0.418 | 0.557 |
| 0.155 | 0.167 | 0.191 | 0.206 | 0.403 | 0.543 | 0.453 | 0.590 |
| 0.152 | 0.165 | 0.192 | 0.208 | 0.400 | 0.540 | 0.443 | 0.581 |
| 0.177 | 0.191 | 0.232 | 0.252 | 0.430 | 0.569 | 0.495 | 0.631 |
| 0.223 | 0.244 | 0.300 | 0.329 | 0.485 | 0.621 | 0.564 | 0.697 |
| 0.259 | 0.286 | 0.281 | 0.310 | 0.527 | 0.662 | 0.541 | 0.674 |

Table 2.1 Mortality population attributable fraction due to insufficient fruit intake by sex and age group

| age | fruit | | fruit(+50g) | | | | fruit(+100g) | | | |
| --- | --- | --- | --- | --- | --- | --- | --- | --- | --- | --- |
|  | male | female | male | % | female | % | male | % | female | % |
| 20-29 | 0.220 | 0.197 | 0.161 | 0.269 | 0.141 | 0.284 | 0.104 | 0.527 | 0.089 | 0.547 |
| 30-39 | 0.214 | 0.193 | 0.155 | 0.274 | 0.136 | 0.296 | 0.100 | 0.535 | 0.083 | 0.572 |
| 40-49 | 0.215 | 0.186 | 0.156 | 0.272 | 0.131 | 0.297 | 0.100 | 0.533 | 0.081 | 0.565 |
| 50-59 | 0.193 | 0.154 | 0.137 | 0.289 | 0.104 | 0.326 | 0.086 | 0.557 | 0.061 | 0.608 |
| 60-69 | 0.146 | 0.110 | 0.099 | 0.325 | 0.069 | 0.377 | 0.058 | 0.601 | 0.037 | 0.662 |
| 70- | 0.118 | 0.111 | 0.076 | 0.355 | 0.070 | 0.367 | 0.043 | 0.637 | 0.039 | 0.647 |

Table 2.2 Mortality population attributable fraction due to insufficient fruit intake by sex and age group, 95%CI

| fruit(+50g)-MPAF | | | | fruit(+100g)-MPAF | | | |
| --- | --- | --- | --- | --- | --- | --- | --- |
| male | | female | | male | | female | |
| lower | upper | lower | upper | lower | upper | lower | upper |
| 0.252 | 0.286 | 0.265 | 0.304 | 0.459 | 0.596 | 0.479 | 0.616 |
| 0.257 | 0.292 | 0.275 | 0.317 | 0.466 | 0.604 | 0.504 | 0.640 |
| 0.255 | 0.289 | 0.276 | 0.318 | 0.464 | 0.602 | 0.497 | 0.633 |
| 0.269 | 0.310 | 0.299 | 0.354 | 0.488 | 0.625 | 0.540 | 0.675 |
| 0.296 | 0.355 | 0.335 | 0.420 | 0.534 | 0.669 | 0.594 | 0.731 |
| 0.315 | 0.394 | 0.324 | 0.410 | 0.568 | 0.705 | 0.579 | 0.716 |

Table 3. 1 Prevalence population attributable fraction due to insufficient vegetable intake by sex and age group

| age | vegetable | | vegetable(+70g) | | | | vegetable(+140g) | | | |
| --- | --- | --- | --- | --- | --- | --- | --- | --- | --- | --- |
|  | male | female | male | % | female | % | male | % | female | % |
| 20-29 | 0.046 | 0.051 | 0.030 | 0.351 | 0.034 | 0.328 | 0.016 | 0.640 | 0.068 | 0.609 |
| 30-39 | 0.043 | 0.045 | 0.027 | 0.369 | 0.029 | 0.354 | 0.015 | 0.661 | 0.063 | 0.654 |
| 40-49 | 0.040 | 0.045 | 0.025 | 0.366 | 0.029 | 0.356 | 0.014 | 0.659 | 0.062 | 0.655 |
| 50-59 | 0.039 | 0.038 | 0.025 | 0.366 | 0.023 | 0.387 | 0.013 | 0.662 | 0.046 | 0.697 |
| 60-69 | 0.033 | 0.030 | 0.020 | 0.382 | 0.017 | 0.425 | 0.011 | 0.674 | 0.029 | 0.736 |
| 70- | 0.034 | 0.036 | 0.021 | 0.373 | 0.023 | 0.356 | 0.012 | 0.656 | 0.030 | 0.643 |

Table 3.2 Prevalence population attributable fraction due to insufficient vegetable intake by sex and age group, 95% CI

| vegetable(+70g)-PPAF | | | | vegetable(+140g)-PPAF | | | |
| --- | --- | --- | --- | --- | --- | --- | --- |
| male | | female | | male | | female | |
| lower | upper | lower | upper | lower | upper | lower | upper |
| 0.251 | 0.451 | 0.243 | 0.412 | 0.561 | 0.719 | 0.534 | 0.684 |
| 0.263 | 0.475 | 0.255 | 0.453 | 0.580 | 0.741 | 0.576 | 0.732 |
| 0.254 | 0.479 | 0.257 | 0.454 | 0.577 | 0.740 | 0.577 | 0.734 |
| 0.251 | 0.481 | 0.268 | 0.505 | 0.580 | 0.744 | 0.612 | 0.783 |
| 0.240 | 0.524 | 0.261 | 0.590 | 0.583 | 0.765 | 0.633 | 0.838 |
| 0.237 | 0.510 | 0.233 | 0.480 | 0.568 | 0.744 | 0.558 | 0.727 |

Table 4.1 Mortality population attributable fraction due to insufficient vegetable intake by sex and age group

| age | vegetable | | vegetable(+70g) | | | | vegetable(+140g) | | | |
| --- | --- | --- | --- | --- | --- | --- | --- | --- | --- | --- |
|  | male | female | male | % | female | % | male | % | female | % |
| 20-29 | 0.118 | 0.133 | 0.078 | 0.342 | 0.091 | 0.317 | 0.044 | 0.627 | 0.055 | 0.588 |
| 30-39 | 0.112 | 0.119 | 0.071 | 0.361 | 0.078 | 0.341 | 0.039 | 0.649 | 0.044 | 0.632 |
| 40-49 | 0.104 | 0.118 | 0.067 | 0.357 | 0.078 | 0.341 | 0.037 | 0.647 | 0.043 | 0.633 |
| 50-59 | 0.103 | 0.101 | 0.066 | 0.359 | 0.063 | 0.371 | 0.036 | 0.651 | 0.033 | 0.675 |
| 60-69 | 0.087 | 0.080 | 0.054 | 0.377 | 0.047 | 0.408 | 0.029 | 0.666 | 0.023 | 0.712 |
| 70- | 0.090 | 0.096 | 0.057 | 0.369 | 0.062 | 0.354 | 0.032 | 0.648 | 0.035 | 0.635 |

Table 4.2 Mortality population attributable fraction due to insufficient vegetable intake by sex and age group, 95%CI

| vegetable(+70g)-MPAF | | | | vegetable(+140g)-MPAF | | | |
| --- | --- | --- | --- | --- | --- | --- | --- |
| male |  | female |  | male |  | female |  |
| lower | upper | lower | upper | lower | upper | lower | upper |
| 0.318 | 0.367 | 0.296 | 0.338 | 0.560 | 0.694 | 0.520 | 0.655 |
| 0.335 | 0.388 | 0.316 | 0.365 | 0.582 | 0.716 | 0.565 | 0.699 |
| 0.328 | 0.387 | 0.316 | 0.366 | 0.580 | 0.714 | 0.566 | 0.699 |
| 0.330 | 0.388 | 0.341 | 0.401 | 0.584 | 0.718 | 0.608 | 0.741 |
| 0.341 | 0.413 | 0.368 | 0.449 | 0.598 | 0.733 | 0.645 | 0.779 |
| 0.335 | 0.403 | 0.322 | 0.385 | 0.581 | 0.715 | 0.568 | 0.702 |

Table 5. CVD disease burden due to insufficient fruit and vegetable intake of male in 2015

|  | stroke | SD | CHD | SD |
| --- | --- | --- | --- | --- |
| original DALYs | 744054 | 1532 | 583146 | 1325 |
| DALYs caused by vegetables | 60726 | 52 | 50800 | 44 |
| DALYs caused by fruits | 100004 | 131 | 83502 | 116 |
| DALYs caused by vegetables (+70g) | 38700 | 33 | 32349 | 28 |
| DALYs caused by fruits (+50g) | 68489 | 93 | 57107 | 84 |
| DALYs caused by vegetables (+140g) | 21295 | 18 | 7978 | 40 |
| DALYs caused by fruits (+100g) | 40469 | 54 | 34007 | 49 |

Table 6. CVD disease burden due to insufficient fruit and vegetable intake of female in 2015

|  | stroke | SD | CHD | SD |
| --- | --- | --- | --- | --- |
| original DALYs | 704244 | 1525 | 357045 | 1003 |
| DALYs caused by vegetables | 59319 | 57 | 31807 | 36 |
| DALYs caused by fruits | 78200 | 110 | 40349 | 79 |
| DALYs caused by vegetables (+70g) | 37981 | 36 | 20340 | 23 |
| DALYs caused by fruits (+50g) | 50936 | 83 | 26004 | 54 |
| DALYs caused by vegetables (+140g) | 21017 | 19 | 5134 | 34 |
| DALYs caused by fruits (+100g) | 28706 | 46 | 14607 | 31 |

Table 7. CVD disease burden due to insufficient fruit and vegetable intake of male in 2030

|  | stroke | SD | CHD | SD |
| --- | --- | --- | --- | --- |
| original DALYs | 946684 | 1820 | 698501 | 1553 |
| DALYs caused by vegetables | 77131 | 64 | 60603 | 52 |
| DALYs caused by fruits | 122350 | 158 | 96409 | 130 |
| DALYs caused by vegetables (+70g) | 49251 | 40 | 38670 | 32 |
| DALYs caused by fruits (+50g) | 82986 | 113 | 65414 | 94 |
| DALYs caused by vegetables (+140g) | 27187 | 22 | 9541 | 47 |
| DALYs caused by fruits (+100g) | 48537 | 67 | 38624 | 55 |

Table 8. CVD disease burden due to insufficient fruit and vegetable intake of female in 2030

|  | stroke | SD | CHD | SD |
| --- | --- | --- | --- | --- |
| original DALYs | 995013 | 1990 | 485838 | 1296 |
| DALYs caused by vegetables | 84441 | 73 | 43774 | 46 |
| DALYs caused by fruits | 108601 | 158 | 54313 | 104 |
| DALYs caused by vegetables (+70g) | 54231 | 47 | 28094 | 29 |
| DALYs caused by fruits (+50g) | 70392 | 115 | 34880 | 69 |
| DALYs caused by vegetables (+140g) | 30212 | 27 | 7001 | 44 |
| DALYs caused by fruits (+100g) | 39507 | 62 | 19545 | 39 |

Table .9 CVD disease burden due to insufficient fruit and vegetable intake of male in 2045

|  | stroke | SD | CHD | SD |
| --- | --- | --- | --- | --- |
| original DALYs | 984318 | 1831 | 700839 | 1465 |
| DALYs caused by vegetables | 79317 | 63 | 60459 | 50 |
| DALYs caused by fruits | 123445 | 157 | 93907 | 124 |
| DALYs caused by vegetables (+70g) | 50658 | 40 | 38589 | 31 |
| DALYs caused by fruits (+50g) | 83245 | 116 | 63271 | 91 |
| DALYs caused by vegetables (+140g) | 28005 | 22 | 9468 | 46 |
| DALYs caused by fruits (+100g) | 48347 | 64 | 37090 | 50 |

Table 10. CVD disease burden due to insufficient fruit and vegetable intake of female in 2045

|  | stroke | SD | CHD | SD |
| --- | --- | --- | --- | --- |
| original DALYs | 1113189 | 2372 | 512225 | 1154 |
| DALYs caused by vegetables | 93860 | 89 | 46424 | 41 |
| DALYs caused by fruits | 119751 | 193 | 56906 | 92 |
| DALYs caused by vegetables (+70g) | 60346 | 56 | 29825 | 27 |
| DALYs caused by fruits (+50g) | 77475 | 137 | 36442 | 64 |
| DALYs caused by vegetables (+140g) | 33718 | 32 | 7310 | 39 |
| DALYs caused by fruits (+100g) | 43387 | 76 | 20381 | 37 |

Table 11. CVD disease burden due to insufficient fruit and vegetable intake of male in 2060

|  | stroke | SD | CHD | SD |
| --- | --- | --- | --- | --- |
| original DALYs | 1000680 | 1992 | 682261 | 1338 |
| DALYs caused by vegetables | 80495 | 68 | 58822 | 46 |
| DALYs caused by fruits | 123398 | 161 | 89978 | 114 |
| DALYs caused by vegetables (+70g) | 51447 | 43 | 37576 | 29 |
| DALYs caused by fruits (+50g) | 82870 | 122 | 60397 | 86 |
| DALYs caused by vegetables (+140g) | 28481 | 23 | 9176 | 43 |
| DALYs caused by fruits (+100g) | 47912 | 66 | 35262 | 47 |

Table 12. CVD disease burden due to insufficient fruit and vegetable intake of female in 2060

|  | stroke | SD | CHD | SD |
| --- | --- | --- | --- | --- |
| original DALYs | 1171262 | 2834 | 528231 | 1189 |
| DALYs caused by vegetables | 98917 | 108 | 48118 | 41 |
| DALYs caused by fruits | 125391 | 229 | 58532 | 90 |
| DALYs caused by vegetables (+70g) | 63672 | 71 | 30951 | 27 |
| DALYs caused by fruits (+50g) | 81024 | 159 | 37439 | 65 |
| DALYs caused by vegetables (+140g) | 35654 | 38 | 7533 | 41 |
| DALYs caused by fruits (+100g) | 45335 | 91 | 20928 | 36 |

**Disability weight (DW) used in the study:**

Table 13. Disability weight of stroke used in the study

| mRS score | Distribution of mRs [1] | Weight [2] | 95% CI | |
| --- | --- | --- | --- | --- |
|  |  |  | low | high |
| 0 | 0.19 | 0 | 0 | 0 |
| 1 | 0.29 | 0.046 | 0.004 | 0.088 |
| 2 | 0.13 | 0.212 | 0.175 | 0.25 |
| 3 | 0.08 | 0.331 | 0.292 | 0.371 |
| 4 | 0.14 | 0.652 | 0.625 | 0.678 |
| 5 | 0.10 | 0.944 | 0.873 | 1 |
| dead | 0.07 | 1 | 1 | 1 |

However, no detailed data of age/gender-specific prevalence cases in different mRS score groups existed. We calculated the sum of products number, and used the number as the DW of stroke: 0.25306 (95%CI: 0.22207~0.28262)

Table 14. Disability weight of coronary heart disease

| **ischemic heart disease [3]** | DW | low | high |
| --- | --- | --- | --- |
| acute myocardial infarction | 0.439 | 0.405 | 0.477 |
| angina pectoris | 0.124 | 0.105 | 0.141 |

[1] Kimura K, Kazui S, Minematsu K, et al. Hospital-based prospective registration of acute ischemic stroke and transient ischemic attack in Japan[J]. Journal of Stroke and Cerebrovascular Diseases, 2004, 13(1): 1-11. <http://www.sciencedirect.com/science/article/pii/S1052305703001381#FIG3>

[2] Hong K S, Saver J L. Quantifying the Value of Stroke Disability Outcomes[J]. Stroke, 2009, 40(12): 3828-3833. <https://www.ncbi.nlm.nih.gov/pmc/articles/PMC2788070/>

[3] World Health Organization. Global burden of disease 2004 update: disability weights for diseases and conditions. <http://www.who.int/healthinfo/global_burden_disease/GBD2004_DisabilityWeights.pdf>

| Table 15. Effects of fruit/vegetable intake on CVD in Japan | | | | | | | |
| --- | --- | --- | --- | --- | --- | --- | --- |
| Journal | Year | Author | Population | Categorize (the first is the reference category) | Multivariate-adjusted HR (95% confidence interval) | Stratified variables |  |
| European Journal of Clinical Nutrition | 2015 | N Okuda et al | 9112 participants aged 30–79 in 1980 | quartiles of FV intake (g/1000 kcal):  men:  113 (0, 137),  154 (137, 170),  190 (170, 213),  249 (213, 576);  women:  148 (11, 178),  201 (178, 225),  251 (225, 282),  332 (282, 748) | adjusted CVD mortality:  0.85 (0.69, 1.05)  **0.72 (0.58, 0.89)**  **0.74 (0.61, 0.91)**  adjusted stroke mortality:  1.10 (0.81, 1.50)  0.83 (0.60, 1.13)  0.80 (0.59, 1.09)  adjusted CHD mortality:  **0.39 (0.23, 0.66)**  0.65 (0.43, 1.00)  **0.57 (0.37, 0.87)** | age, sex, body mass index (kg/m^2^), smoking habit (current-, ex-, never), drinking habit (daily drinkers and others), sodium intake (mg/1000 kcal) and intakes of meat (g/1000 kcal), fish and shellfish (g/1000 kcal), milk and dairy products (g/1000 kcal) and soybeans and legumes (g/1000 kcal) |  |
| Diabetes Care | 2013 | Shiro Tanaka et al | 1,414 patients with type 2 diabetes aged 40–70 years in 1995-1996 | quartiles of intake (g/d): vegetable:  158.6 ± 64.7  258.3 ± 71.2  351.7 ± 86.1  518.3 ± 159.6;  Fruits:  70.1 ± 57.0  113.5 ± 74.0  147.3 ± 86.8  203.1 ± 139.3 | stroke risk:  0.72 (0.36–1.44)  0.45 (0.19–1.04)  **0.35 (0.13–0.96)**  CHD risk:  1.25 (0.66–2.34)  1.34 (0.68–2.63)  0.81 (0.36–1.84) | age, sex, BMI, HbA1c, diabetes duration, diabetic retinopathy, treatment by insulin, treatment by oral hypoglycemic agents, SBP, LDL cholesterol, HDL cholesterol, triglycerides, current smoking, physical activity, alcohol intake, and proportions of total fat, saturated fatty acid, n-6 fatty acid and n-3 fatty acid, dietary cholesterol, and sodium intake; and further adjusted for total energy intake |  |
| American Society for Nutrition | 2013 | Linda M Oude Griep et al | 4680 men and women aged 40–59 y from Japan, China, the UK, and the US. | among East Asian participants raw fruit consumption intake: above median intake or not (50 g/1000 kcal: low: 16±15; high: 106±57. | in participants from Japan and China, BP difference (mm Hg):  systolic: 0.15 (-0.37, 0.67)  diastolic: 0.37, (0.02, 0.71) | adjusted for age, sex, and sample; plus intakes of energy (kcal) and alcohol (g/d), smoking status, years of education, physical activity during leisure time, use of dietary supplements, adherence to any special diet, history of cardiovascular disease or diabetes mellitus, family history of cardiovascular disease, urinary sodium, and use of antihypertensive, cardiovascular disease, or diabetes medication; plus intake of low-fat dairy products, raw and cooked vegetables, fiber-rich cereals and grains, red and processed meats, nuts and seeds, and fish and shellfish; BMI |  |
| European Heart Journal | 2012 | Eri Eguchi et al | 18747 men and 24263 women aged 40–79 without a history of stroke or coronary heart disease (CHD) at baseline in 1988–90 were followed up until 2006. | eat fruits everyday vs others | CVD mortality: 0.93 (0.82–1.06) | age, history of hypertension, history of diabetes, education level, regular employment, perceived mental stress, and seven health behaviours other than specified variable |  |
| J Epudemiol | 2011 | Yamada T et al | from April 1992 through July 1995, 10,623 participants (4147 men, 6476 women) who had no history of CVD or carcinoma were followed during an average period of 10.7 years | frequency of citrus fruit intake: infrequent  1-2/month  1-2/week  3-4/week  almost daily | CVD risk:  men:  0.70 (0.47–1.05)  0.75 (0.52–1.09)  **0.63 (0.41–0.97)**  0.57 (0.33–1.01)  women:  0.83 (0.49–1.41)  0.68 (0.42–1.11)  0.75 (0.46–1.22)  **0.51 (0.29–0.88)** | adjusted for age, study area, body mass index, systolic blood pressure, total cholesterol concentration, physical activity index, smoking status, alcohol consumption, education level, and marital status |  |
| British Journal of Nutrition | 2009 | Junko Nagura et al | 25 206 men and 34 279 women aged 40–79 years in 1988–90 | quartiles of intake (servings/week):  fruit: 0.9, 2.3, 3.9, 5.9; vegetables:1.2, 2.3, 3.4, 5.2 | Stroke mortality:  **0.81 (0.69, 0,96)**  **0.76 (0.64, 0.90)**  **0.65 (0.53, 0.80)**  CHD mortality:  0.97 (0.75, 1.24)  0.84 (0.65, 1.10)  0.79 (0.58, 1.08)  Total CVD mortality:  0.90 (0.80, 1.00)  **0.89 (0.79, 0.99)**  **0.77 (0.67, 0.88)** | sex, age, BMI, smoking status, alcohol intake, hours of walking, hours of sleep, education years, perceived mental stress, cholesterol intake, SFA intake, n-3 fatty acids intake, sodium intake and histories of hypertension and diabetes; and further for fruit/vegetable and bean intakes |  |
| The Journal of Nutrition Nutritional Epidemiology | 2008 | Kozue Nakamura et al | in 1992, 13,355 men and 15,724 women in Takayama, Gifu, Japan | quartiles of intake (servings/d) or (g/d):  men:  fruit:  0.3, 0.7, 1.3, 2.6;  24.1, 62.2, 110.6, 211.7  vegetable:  2.2, 3.4, 4.6, 7.1;  176.4, 263.7, 360.1, 553.6 women:  fruit:  0.4, 0.9, 1.5, 2.7;  35.7, 77.0, 122.5, 213.6  vegetable:  2.5, 3.6, 4.8, 7.4;  195.5, 281.1, 375.6, 573.9 | CVD mortality:  men: fruit:  1.10 (0.70–1.74)  0.93 (0.57–1.52)  1.27 (0.81–2.01)  vegetable:  0.81 (0.46–1.43)  1.08 (0.63–1.85)  1.02 (0.57–1.82)  Women: fruit:  0.95 (0.59–1.52)  0.71 (0.42–1.18)  0.83 (0.51–1.34)  vegetable:  0.84 (0.49–1.45)  0.83 (0.47–1.47)  0.77 (0.41–1.46) | nondietary confounders (age, total energy, marital status, years of education, BMI, smoking status (never, former, current), alcohol intake, exercise, history of hypertension or diabetes mellitus (nondietary factors), and menopausal status) and dietary confounders (total protein, saturated fat, and sodium intake) |  |
| American Journal of Epidemiology | 2008 | Ribeka Takachi et al | during 1995–1998 in nine areas, 77,891 men and women aged 45–74 years | Quartiles of FV intake (g/d): 186, 335, 482, 733 | CVD risk: total FV:  0.90 (0.78, 1.03)  **0.80 (0.69, 0.93)**  **0.76 (0.65, 0.90)**  Fruit:  **0.87 (0.75, 0.99)**  **0.70 (0.60, 0.81)**  **0.70 (0.59, 0.82)**  Vegetables:  0.93 (0.80, 1.07)  **0.81 (0.69, 0.94)**  0.89 (0.77, 1.04) | age (5-year groups), and public health center area; Further adjusted for body mass index in kg/m2 (<19, 19–22.9, 23–26.9, and 27), physical activity in metabolic equivalent task-hours/day (<30, 30–34.9, 35–39.9, and 40), smoking status (never, past, and current), alcohol consumption (none, occasional, 1–149, 150–299, 300–449, and 450 g ethanol/week), quartile of energy intake, screening examination (blood pressure, chest radiograph, gastric photofluorography, gastrointestinal endoscopy, fecal occult blood test, barium enema, colonoscopy, mammography, Papanicolaou smear), medication (hypertension, hyperlipidemia, diabetes mellitus), and daily vitamin supplement use |  |
| Stroke | 2003 | Sauvaget, C. | A prospective cohort study of 40 349 Japanese men and women was initiated in 1980–1981 and followed until 1998 | Frequency of green-yellow vegetables and fruits intake: 0-1/week, 2-4/week, daily | Stoke risk:  men:  0.87 (0.71–1.07)  0.81 (0.63–1.03)  women:  1.00 (0.85–1.18)  0.85 (0.70–1.02) | age-stratified, and adjusted for radiation dose, city, BMI, smoking status, alcohol habits, education level, medical history of hypertension, myocardial infarction, diabetes, and consumption of animal products (egg, dairy, fish) |  |

(Figure 1.1-1.4 were done by JMP 13.0)


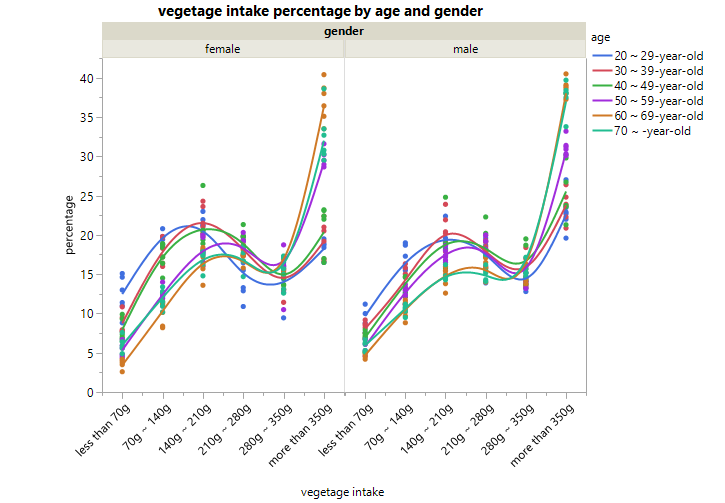


**Figure 1.1**


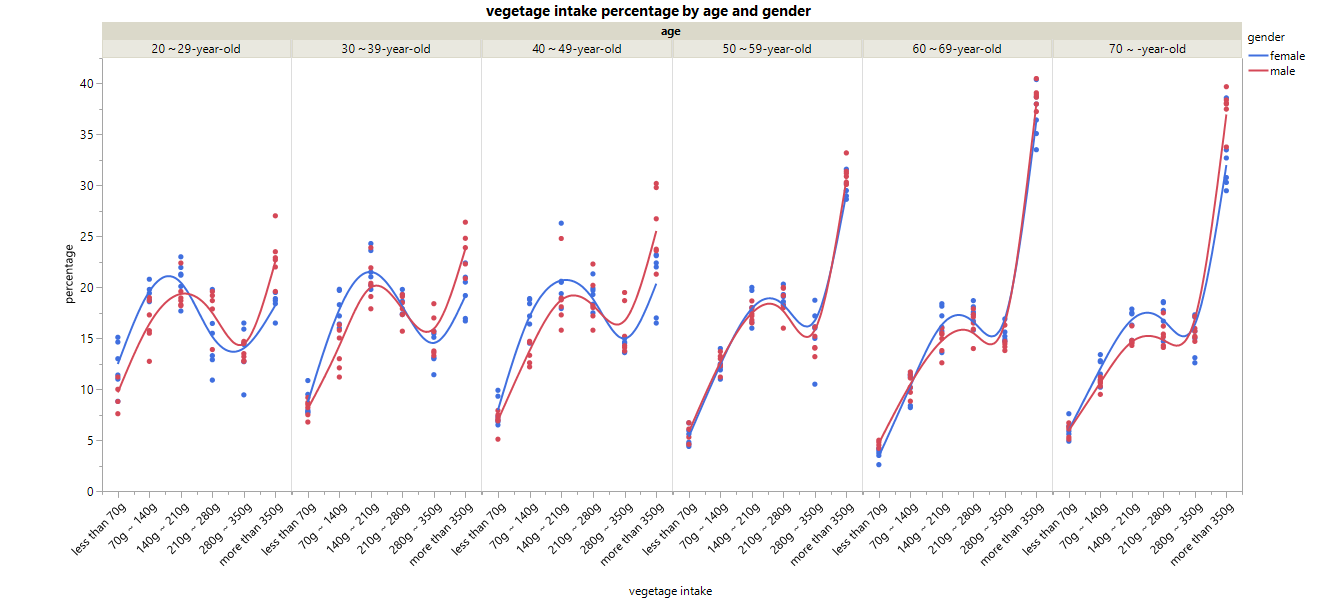


**Figure 1.2**


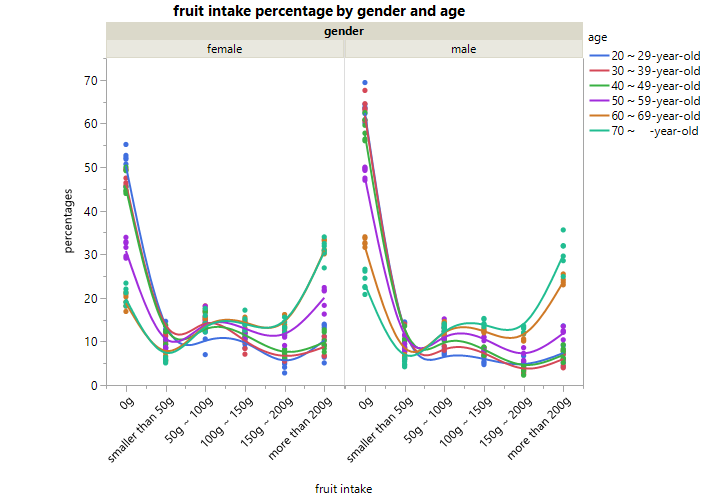


**Figure 1.3**


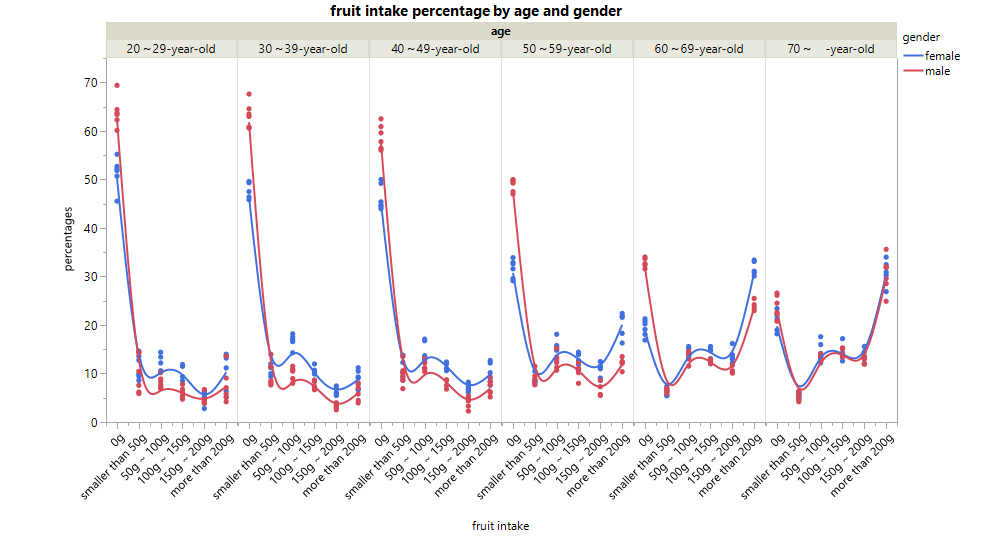


**Figure 1.4**
